# Correlation and comparison of Risser sign versus bone age determination (TW3) between children with and without scoliosis in Korean population

**DOI:** 10.1186/1749-799X-4-36

**Published:** 2009-09-20

**Authors:** Hitesh N Modi, Chetna H Modi, Seung Woo Suh, Jae-Hyuk Yang, Jae-Young Hong

**Affiliations:** 1 Scoliosis Research Institute, Department of Orthopedics, Korea University Guro Hospital, Seoul, Korea

## Abstract

**Background:**

Most studies comparing the Risser staging for skeletal maturity are representing the American or European standards which are not always applicable to Asian population who have relatively less height and body mass. There is no article available that compares the Risser sign and bone age correlation between patients with idiopathic scoliosis and patients without scoliosis.

**Materials and methods:**

To analyze and compare the skeletal age with the Risser sign between scoliosis and non-scoliosis group, a cross-sectional study was done in 418 scoliosis (untreated, bracing or surgically) and 256 non-scoliosis children of Korean origin. Relationship was found in both groups using Pearson correlation test.

**Results:**

In scoliosis group, Pearson correlation exhibited significant correlation (p < 0.01) between Risser sign and chronological age (r^2 ^= 0.791 for girls, 0.787 for boys) and Risser sign and TW3 age (r^2 ^= 0.718 for girls, 0.785 for boys). Non-scoliosis group also showed significant relationship (p < 0.01) between Risser sign and chronological age (r^2 ^= 0.893 for girls, 0.879 for boys) and Risser sign and TW3 age (r^2 ^= 0.913 for girls, 0.895 for boys). Similarly, comparing Cobb angles of each patient according to their Risser staging, exhibited that if scoliosis remains untreated Cobb angle will increase with the increase in their Risser staging (r^2 ^= 0.363 for girls, 0.443 for boys; p < 0.01).

**Conclusion:**

Our results showed that chronological age is equally as reliable as skeletal age method to compare with Risser sign, and therefore, we do not mean to imply that only the Risser sign compared with skeletal age should be considered in the decision making in idiopathic as well as non-scoliosis patients of Korean ethnicity. Concomitant indicators such as menarchal period, secondary sex characteristics, and recent growth pattern will likely reinforce our data comparing Risser sign with skeletal age in decision making.

## Introduction

Prognostic studies related to the risk of curve progression in patients with idiopathic scoliosis (IS) have been hampered by poor measurements of skeletal maturity and arbitrary definitions of curve progression. Literature [1] described a number of potential skeletal maturity indicators, including Risser sign, the Oxford stage, The Greulich and Pyle (GP) and Tanner-Whitehouse-III (TW3) maturity assessments, and a number of serological skeletal maturity markers in girls with idiopathic scoliosis. Using the hand-wrist radiographs, there are two possible analysis schemes are defined for the TW3 method. The first one, named RUS, uses 13 bones (the phalanges, radius, and ulna). The other one uses 20 bones (the 13 bones previously defined and the 7 bones of the carpal region). We have chosen to build a method based on the 13 RUS bones, because the carpal region is not valid after a certain age [2]. Tanner-Whitehouse composite scores are based on osseous stages and events at each level. The Tanner-Whitehouse method involves measuring individual bone parts to create an index value which was matched with prepared table for both male and females to find out their bone age. Senders et al [1] identified that Tanner-Whitehouse-III RUS skeletal maturity assessment [2,3] method is the most closely related to curve behavior, or, more specifically, to the timing of curve take-off in early adolescence, which is termed the curve acceleration phase (CAP). Similarly Wang et al [4] proved that digital bone age assessment (DSA) is a reliable indicator for predicting residual spinal growth potential in idiopathic scoliosis patients, but it should be correlated with menarchal status and chronological ages.

Assessing the skeletal maturity in patients with adolescent idiopathic scoliosis often gives the guideline for the treatment regarding bracing weaning time or surgical consideration. Since chronological age is not an accurate predictor of the maturity, there are several methods have been described in the literature to find out skeletal maturity. However, none of those is proved to be accurate enough to predict the residual growth potential of the spine in idiopathic scoliosis. Risser sign is used most frequently to assess the growth of the patient in relation to clinical problems since it was described by Risser in 1958[5]. Although the Risser sign is still an acceptable prognostic sign in the evaluation of IS patients, there is considerable controversy about its reliability for predicting the cessation of vertebral growth [6-11]. Binodi et al have correlated Risser sign with bone age in 111 patients with adolescent idiopathic scoliosis for assessing the maturity [7]. This study did not have enough number of patients in all groups, and they used the Greulich-Pyle method for bone age assessment. Additionally most of the studies are representing the American or European standards which we think not always applicable to Asian population who have relatively less height and body mass. There is no article available that compares the Risser sign and bone age correlation between patients with idiopathic scoliosis and patients without scoliosis.

In present paper, we correlated Risser sign with chronological and skeletal age measured with Tanner-Whitehouse-III method in our study population with Korean ethnicity. Our aim was to establish the skeletal average age, both in male and females that correlates with the Risser sign the most. In addition we also compared this correlation between the patients with idiopathic scoliosis and without scoliosis to find out if there is any difference exists.

## Materials and methods

We have analyzed skeletal age and Risser sign with their Cobb angle in 418 patients with idiopathic scoliosis (IS) and 256 patients without scoliosis (control). All the patients with scoliosis and without scoliosis were grouped into group1 and group2, respectively. All patients selected for this study had an age between minimum 9 and maximum 16 years for girls, and minimum 9 and maximum 17 years for boys. Average age was 13.52 years (range, 9-17) and 13.97 years (range, 9.1-17) for group1 and 2, respectively. There were 95 boys and 323 girls in group1 and 140 boys and 116 girls in group2. Bone age determination was done by analyzing left hand posteroanterior (PA) radiogram using TW3 [3] method. All radiograms were taken after obtaining an informed consent from patients and their parents and explaining them the purpose of our study. Specific care was taken that in both groups the date of wrist radiogram, pelvis radiogram (showing Risser sign) and spine (showing Cobb angle) were the same to prevent any biased result. Those radiograms that did not fulfilled all these criteria were excluded from the study. All radiograms were taken by a single radiologist on single radiogram machine to avoid errors regarding patient positioning and distance from the X-ray tube. All patients in group1 did not have any bracing or operative treatment while taking the radiogram and calculating Cobb angle. Risser sign represents progressive maturation of the iliac crest apophysis [5]. In the AP radiographic projection of the pelvis, Risser sign was evaluated for all patients. Similarly, Cobb angle for the patients in group1 was calculated using Cobb method on AP radiogram of whole spine. All skeletal age assessment, Cobb angle measurement and Risser sign evaluation were done separately by two fellows who were familiar with all measurement techniques. All measurements were done on stored digital radiograms using picture-archiving and communication system (PACS).

Skeletal age was subsequently compared with iliac crest apophysis maturation (Risser sign) to determine the reliability of Risser sign as it correlates to the skeletal age determination by TW3 method. Similarly, chronological age were also compared with the Risser staging and Pearsons correlation coefficient graph was plotted to find out any difference from chronological age to TW3 bone age. Comparison was done in both the groups to find out any difference in patients with scoliosis from in patients without scoliosis. Additionally skeletal age and Risser sign were also compared according to severity of curve and gender in entire patients group to find any relation. Linear regression curve was plotted to compare various variables with the Risser sign and skeletal age assessment.

## Results

**Figure 1:**
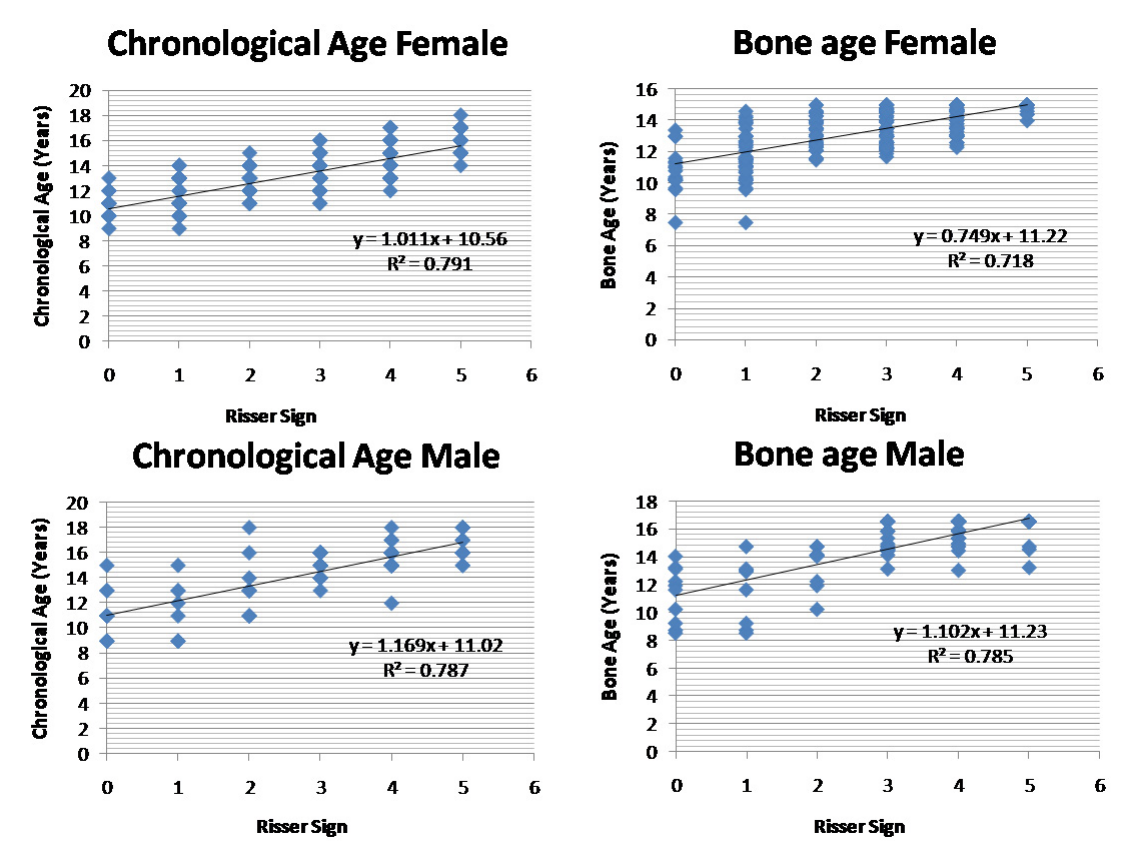
**Correlation of the skeletal age and bone age (TW3 method) were plotted against the Risser sign for girls and boys separately in idiopathic scoliosis group (group1)**. Values r^2 ^denotes the Pearson correlation coefficient value that suggested that TW3 bone age measurement is equally reliable with chronological age while comparing with the Risser sign for skeletal maturity.

**Figure 2:**
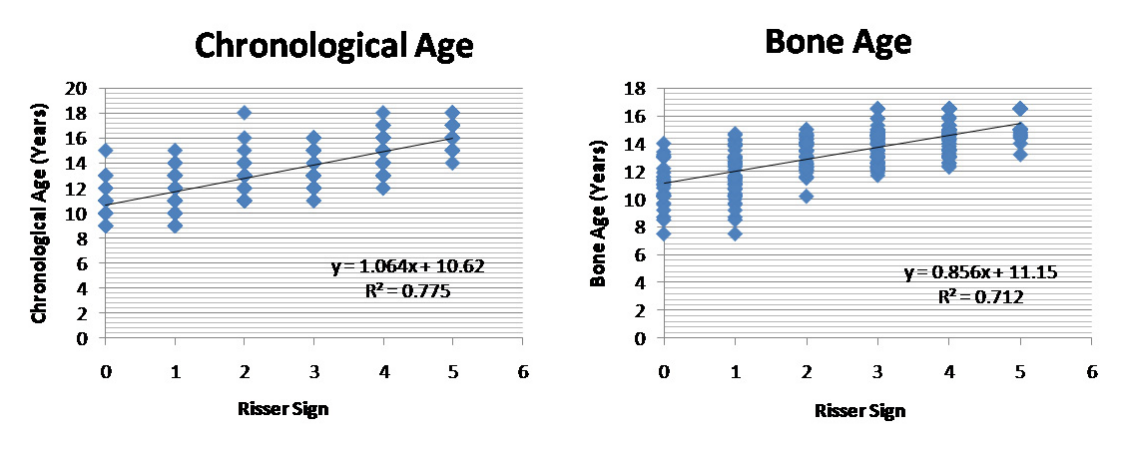
**Correlations of the skeletal age and bone age (TW3 method) were plotted against the Risser sign for combined population in idiopathic scoliosis group (group1)**. Values r^2 ^denotes the Pearson correlation coefficient value that suggested that TW3 bone age measurement is equally reliable with chronological age while comparing with the Risser sign for skeletal maturity.

**Figure 3:**
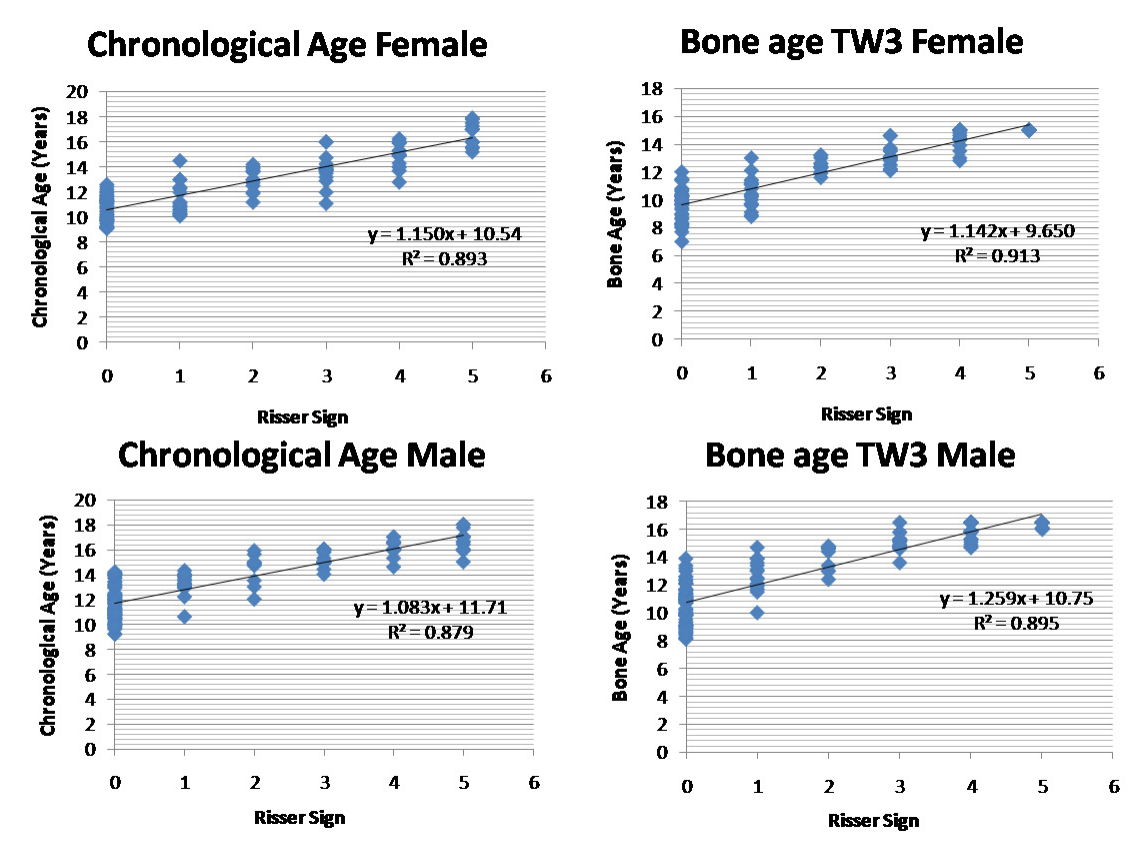
**Correlations of the skeletal age and bone age (TW3 method) were plotted against the Risser sign for girls and boys separately in non-scoliosis group (group2)**. Values r^2 ^denotes the Pearson correlation coefficient value that suggested that TW3 bone age measurement is equally reliable with chronological age while comparing with the Risser sign for skeletal maturity.

**Figure 4:**
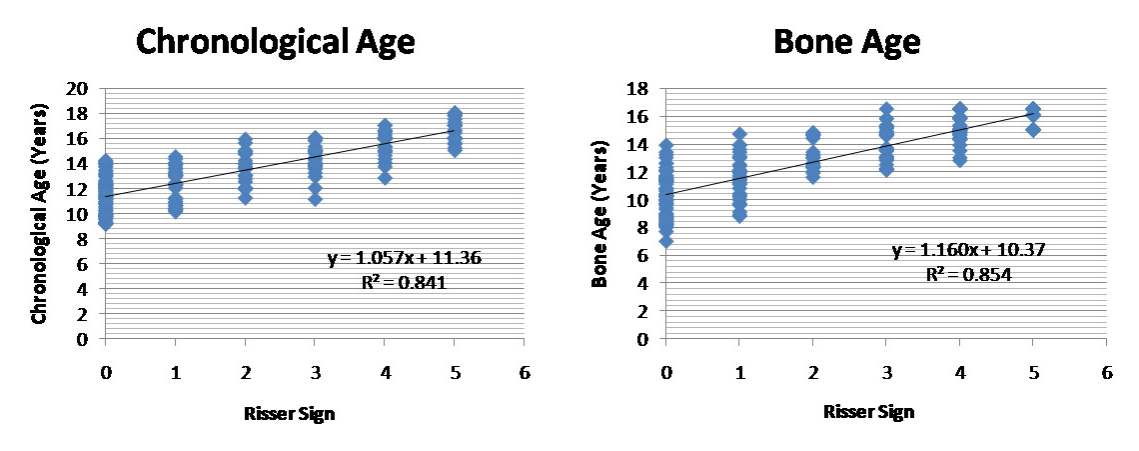
**Correlations of the skeletal age and bone age (TW3 method) were plotted against the Risser sign for combined population in non-scoliosis group (group2)**. Values r^2 ^denotes the Pearson correlation coefficient value that suggested that TW3 bone age measurement is equally reliable with chronological age while comparing with the Risser sign for skeletal maturity.

**Figure 5:**
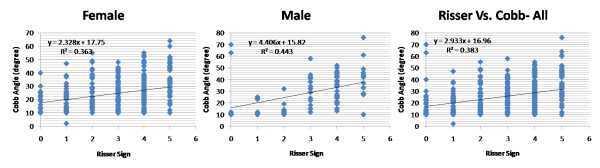
**correlation of the Cobb angle was plotted against the Risser sign for girls and boys separately and for combined population in idiopathic scoliosis group (group1)**. Values r^2 ^denotes the Pearson correlation coefficient value that suggested that if idiopathic scoliosis children were remained untreated, their Cobb angles would likely to increase with the increase in the Risser sign.

Table 1 shows average chronological and TW3 skeletal age, and numbers of patients having Risser staging 1 to 5 respectively in group1 along with their average Cobb angles. Analyzing our results using Pearson correlation coefficient test, it showed value r^2 ^= 0.791 and 0.718 while comparing Risser sign with chronological and TW3 age, respectively in girls with scoliosis; and it was found to be r^2 ^= 0.787 and 0.785 for boys with scoliosis. Similarly in group2, results showed value r^2 ^= 0.893 and 0.913 for girls; and 0.879 and 0.895 for boys, comparing bone age versus Risser sign and Chronological age versus Risser sign, respectively. Our results showed that TW3 method is as reliable as chronological age to compare with Risser sign in both the groups. When all values were compared combined for both genders, r^2 ^= 0.775 and 0.712 for chronological and bone age, respectively in group1 and r^2 ^= 0.841 and 0.854 in group2 which suggested that both methods are equally reliable to correlate the Risser sign in scoliosis as well as non-scoliosis patients to consider the skeletal maturity. Additionally comparing the average chronological age with the average bone age according to the Risser sign in group1, there was no significant difference found for girls (p = 0.91), for boys (p = 0.98) and combined (p = 0.93) in patients with scoliosis (Fig 1, 2). Similarly in group2 it also did not exhibit any significant difference for girls (p = 0.49), boys (p = 0.69) and combined (0.44) population which showed that average values of bone age in each patient is the same with their chronological age in these population (Fig 3, 4). Additionally when we compared the Cobb angles of each patient according to their Risser staging using Pearson correlation coefficient r^2 ^found to be 0.363, 0.443 and 0.383 for girls, boys and combined population, respectively which exhibited that if scoliosis remains untreated Cobb angle will increase with the increase in their Risser staging (Fig 5).

**Table 1:** Values of TW3 bone age and chronological age according to their Risser staging with their Cobb angle in idiopathic scoliosis patients (group1).

**Sex**	**Risser Stage**	**No of Pt**	**Age**	**SD**	**r^2 ^value**	**Bone age**	**SD**	**r^2 ^value**	**Cobb**	**SD**	**r^2 ^value**
**Female**	0	17	10.76	1.03	0.791*	10.86	1.43	0.718*	19.41	8.12	0.363*
	1	44	11.72	1.35		11.81	1.51		20.22	8.73	
	2	44	12.72	0.97		13.26	0.96		24.72	11.69	
	3	85	13.31	1.05		13.45	0.93		23.34	9.62	
	4	93	14.47	1.03		14.16	0.85		24.43	11.47	
	5	40	16.15	0.86		14.93	0.21		34.95	12.8	

**Male**	0	10	11.2	1.98	0.787*	11.26	1.98	0.785*	21.9	23.57	0.443*
	1	8	11.37	2.26		11.46	2.36		15.75	6.88	
	2	9	13.33	2.39		13.33	1.54		15.22	6.79	
	3	19	15	0.94		15.1	0.97		31	13.16	
	4	33	15.69	0.98		15.88	0.86		34.03	10.79	
	5	16	16.62	1.02		16.05	0.99		38.06	16.53	

r^2 ^values are explained by Pearson correlation coefficient test.

* Suggestive of p value < 0.01.

## Discussion

The Risser sign has been and is still used commonly by orthopedic surgeons as a guide to the initiation or cessation of brace treatment, and the indication for surgery [12,13]. The reason for using the Risser sign as a measure of skeletal maturity is that the chronological age of the patient is assumed to be inaccurate. Additionally it is readily available on good anteroposterior standing radiogram of spine while evaluating the patients with scoliosis. Therefore it is better to correlate the reliability of Risser sign with skeletal age to find out the average skeletal age of the Risser sign in the population. Most literature correlated the skeletal age with the Risser sign in American or European children, however, no literature has been reported for Asian population in which comparing the Risser sign with skeletal age is not reliable. The purpose of this study was to evaluate the correlation between the Risser sign with skeletal age measured by TW3 method, and also to compare the reliability of skeletal age with that of chronological age in Korean population. Using the control group in our study we also aimed at to find out the difference, if exists, the appearance of the Risser sign and skeletal age in scoliosis patients from control patients (without scoliosis) of the same ethnicity. We believe that this is the first study comparing such difference among scoliotic and non-scoliotic groups.

Binodi et al [7] found that the correlation coefficient of the Risser stage versus skeletal age was 0.56 for girls while Scoles et al [10] found it to be 0.81 for Risser stage versus chronological age. Thus they using these methods, chronological age provides a more accurate measure of skeletal age than the Risser sign. Both of these studies were done using Greulich Pyle method. While Dhar et al [8] compared Risser stages with skeletal age using TW2 atlas [14]. Here in present study we have used TW3 method to find out relationship between the Risser sign and skeletal age in Korean population. We also tried to estimate general appearance of the Risser sign according to skeletal age in Korean population aiming to make a baseline data for this population. We found that the comparison of the Risser staging versus chronological age and the Risser staging versus TW3 bone age in both groups (with scoliosis and without scoliosis) did not differ from each other. Our study proved that that there is no difference on appearance of the Risser sign based on the presence or absence of idiopathic scoliosis in Korean population. We, however, agree that the correlation of Risser sign with chronological and TW3 bone age in non-scoliosis group had a little higher reliability than that of scoliosis group. However, this difference of the chronological and TW3 bone age was not statistically significant between group 1 and 2 (p > 0.05).

Assessment of skeletal maturity is necessary in the management of children with skeletal growth abnormalities and in the decision making of the other deformities like scoliosis. It is quite difficult to determine developmental maturation of children from statural height, weight and chronological age [15]. Recently Wang et al [4] compared histological examination of superior and inferior endplates of vertebral body and compared them to digital skeletal age (DSA) to identify residual growth potential of spinal column in 39 Chinese girls. Their histological grading of the endplates showed that DSA is a more reliable indicator than menarchal status or chronological age for predicting the spinal residual growth potential in idiopathic scoliosis patients. However, histological grading is not possible in routine clinical practice, rather impractical to find out maturity level. And as per their conclusion, we also used DSA to identify skeletal maturity and that was compared with the Risser sign to establish the average range of DSA using TW3 method in Korean population. Additionally we have compared both genders in our study in both groups with and without scoliosis, and we found that the range and the mean age for the appearance of the Risser sign were similar in boys and girls of both the groups.

In present study we have found that average chronological age and skeletal age were with their standard deviations in both groups as described in table 1 and 2, respectively. Little and Sussman [9]used TW2 method for skeletal age measurement as they found errors by estimating with Grelich-Pyle atlas as mentioned that an error of ± 8 months is possible in correctly assessing skeletal age [16]. While Cundy et al [17] noted that 10% of children were assigned skeletal ages varying by >2 years. However, this was not the purpose of our study. We were willing to establish the average skeletal age and their range using TW3 method, by which the different stages of the Riser would appear in scoliosis as well as non-scoliosis patient groups. From our results we could see that in scoliosis group, the average skeletal age would vary within standard deviation of less than one year for girls and less than 1.5 years in boys; while the chronological age would vary within standard deviation of more than one year for girls and more than 1.5 years for boys. Similarly, for non scoliosis group skeletal age range varied less than 1 year's standard deviation for both genders while it varied more than 1 year standard deviation using chronological age. So this pointed out that although there is no significant difference of comparing the Risser sign with skeletal or chronological age, skeletal age method would be better due to it lower range in each Risser stage. Second important thing to be noted that average skeletal age according to the Risser staging was slightly higher than chronological age in group1 while it was slightly lower in group2. This might be an important difference between the patients with scoliosis and without scoliosis. However, this is a cross sectional study; so we would require a longitudinal study to establish if really such difference exists in Korean population. Furthermore, no data in the literature support the use of the Risser sign as an accurate estimation of the skeletal maturity. We in our study propose that the Risser sign should also be matched with the skeletal age as we mentioned before considering the skeletal maturity to proceed with treatment in scoliosis group or non-scoliosis group.

**Table 2:** Values of TW3 bone age and chronological age according to their Risser staging in non-scoliosis patients (group2).

**Sex**	**Risser Stage**	**No of Pt**	**Age**	**SD**	**r^2 ^value**	**Bone age**	**SD**	**r^2 ^value**
**Female**	0	36	10.68	1.01	0.893*	9.63	1.17	0.913*
	1	19	11.4	1.22		10.53	1.1	
	2	12	12.97	0.94		12.45	0.5	
	3	15	13.77	1.15		13.11	0.77	
	4	18	15.07	0.98		14.44	0.71	
	5	16	16.55	0.86		15	0	

**Male**	0	63	11.55	1.36	0.879*	10.5	1.45	0.895*
	1	15	13.23	0.91		12.64	1.18	
	2	10	14.43	1.21		13.96	0.9	
	3	16	15.25	0.61		15.05	0.64	
	4	19	16.1	0.55		15.85	0.67	
	5	17	16.72	0.79		16.42	0.17	

r^2 ^values are explained by Pearson correlation coefficient test.

* Suggestive of p value < 0.01.

There were few lacunae in our study. First one is that this study is not a longitudinal study but a cross sectional study in 418 idiopathic scoliosis and 256 non-scoliosis patients. Additionally potential errors in skeletal maturation inherently relate to race, hormonal levels, genetic background, and nutrition. However, we believe that our study was done in a large number of children in Korean population with the same ethnicity. Therefore our data would provide important information regarding the skeletal maturity in this population which is different than American and European ethnicity showing the relationship of Risser sign with skeletal age only. While our data shows that chronological age is equally as reliable as skeletal age method to compare with the Risser sign, and therefore, we do not mean to imply that only the Risser sign compared with skeletal age should be considered in the decision making in idiopathic as well as non-scoliosis patients of Korean ethnicity. Concomitant indicators such as menarchal period, secondary sex characteristics, and recent growth pattern will likely reinforce our data comparing the Risser sign with skeletal age in decision making.

## Competing interests

The authors declare that they have no competing interests. Each author certifies that he has no commercial associations (e.g. consultancies, stock ownership, equity interests, patent/licensing arrangements, etc) that might pose a conflict of interest in connection with the submitted article.

## Authors' contributions

HNM and CHM have contributed in conception and design of data, analysis and interpretation of data, drafting the manuscript and revising it critically, SWS has contributed in conception and design of data, drafting the manuscript and given the final approval of manuscript, IWS has contributed in drafting the manuscript and data analysis, JHY has contributed acquisition of data and revising it critically, and JYH has contributed in drafting the manuscript and designing of data and revising it critically. All authors read and approved the final manuscript.
